# Transformations in doctor–patient responsibilities in China’s quasi-marketised healthcare system

**DOI:** 10.1186/s12939-025-02690-1

**Published:** 2025-11-13

**Authors:** Haoyang Liu, Alan Walker

**Affiliations:** 1 School of Sociological Studies, Politics and International Relations, The University of Sheffield, Sheffield, United Kingdom; 2 Social Policy & Social Gerontology, School of Sociological Studies, Politics and International Relations, The University of Sheffield, Sheffield, United Kingdom

**Keywords:** Healthcare, Marketisation, Privatisation, Doctor-patient responsibilities, China

## Abstract

**Background:**

Following a series of systemic reforms, China’s healthcare system now takes a quasi-marketised form, with an uneasy combination of state regulation and market mechanisms, which has fundamentally reshaped the distribution of responsibilities between doctors and patients. This study employs institutional theory to analyse the institutional factors in shaping the doctor-patient responsibilities within the current healthcare system.

**Methods:**

This qualitative study involved thematic analysis of semi-structured interviews with 28 doctors and patients from various provinces in China. Participants were purposively selected to reflect diverse experiences across healthcare settings. Thematic analysis was conducted to identify and interpret key patterns within the data.

**Results:**

Three main dimensions of privatisation emerged from the analysis: accessing healthcare, care coordination, and healthcare financing. Findings indicate that marketisation has significantly increased the responsibilities placed on individual doctors and patients, effectively transferring systemic burdens to these individuals. Doctors face intensified pressures to manage care within fragmented health services, while patients confront greater personal responsibility in navigating access to care, coordinating their treatments, and handling healthcare expenses.

**Conclusions:**

This study extends the application of institutional theory to the healthcare context. It demonstrates the regulative, normative, and cultural–cognitive dimensions of the healthcare system in shaping and constructing of doctor–patient responsibility. The concept of privatised responsibilities proposed here provides a useful theoretical lens for analysing the effects of quasi-marketisation and offers a foundation for future research on doctor-patient responsibility and accountability in healthcare systems.

## Introduction

Historically, medicine and society adhered to the ethical norm that a physician’s primary duty is to the patient’s welfare [1]. Here, one of key features of the doctor-patient responsibility is a client centred ideology where doctors are seen to act in the best interests of patients, which forms the foundation of trust [2]. Scholars have proposed various models of doctor-patient responsibilities, primarily based on the model of professional-dominated and patient-centred care, largely focusing on the interpersonal and role-based distribution of responsibility within clinical encounters in Western context [1, 3–5]. Less is known about the impact of institutional and systemic transformations in reshaping the doctor-patient responsibilities in the Global South or in such a system that has undergone systematic change in recent years.

China provides an ideal context for examining this issue. Since its establishment in the 1950s, the Chinese healthcare system has shifted from a collective welfare structure in which medical services were largely state funded to the market-driven one, which sought to introduce competition and financial incentives by allowing hospitals to retain profits and requiring them to become financially self-sustaining [6]. Although marketisation is generally believed to encourage efficiency and communication [5], the marketised healthcare reform transformed professional and moral relationships into transactional ones without adequate institutional safeguards [7]. The withdrawal of state funding and the profit-oriented incentive intensified patients’ economic burdens, eroded interpersonal trust among doctors and patients, turning marketisation into a source of doctor–patient conflicts rather than improved communication [4, 7, 8]. By the early 2000s, this full marketisation model was widely viewed as unsuccessful and the government introduced the “new healthcare reform” in 2009, marking a shift toward a quasi-market system that combines state regulation with market mechanisms [7]. It re-emphasised equity, universal coverage, and public responsibility, while maintaining certain efficiency-driven incentives.

Contrary to the expectation that the reforms would improve healthcare quality, access to medical resources has significantly deteriorated, leaving a critical gap between policy intentions and outcomes [8]. The proportion of personal health expenditure rose from 361.9 yuan (approximately £40.2) in 2000 to 6425.3 yuan (approximately £713.9) in 2024, reflecting an average annual growth rate of 12.73%. Meanwhile, out-of-pocket spending accounted for 27.7%, exceeding the 15–20% range recommended by the World Health Organization [9, 10]. Doctors as frontline medical providers have gradually faced criticism for being profit driven. Medical disputes have continued to escalate. Hospitals in some regions reported that 89.84% of emergency department doctors have experienced verbal abuse, insults, threats, or even physical violence [11], which highlights the increasing tensions in doctor-patient relationships. Dominant perspectives within health policy, epidemiological and biomedical frameworks prioritise clinical outcomes but overlook how healthcare systems structure responsibility between doctors and patients [12].

This study therefore analyses the institutional norms in shaping doctor–patient responsibilities. It contributes to the theoretical understanding of institutional norms in healthcare, enriches empirical knowledge in doctor–patient responsibilities within the transformations, reflecting the changed responsibilities under quasi-market conditions.

The first of the following five sections summarises the literature on the context of healthcare reforms in China and the changed doctor–patient responsibilities. The second reviews the literature on institutional change, which serves as the theoretical foundation of the study. The third section describes the research methods, detailing a qualitative approach based on interviews with 28 frontline doctors and their patients who were directly engaged in the current quasi-marketised healthcare system. The analysis demonstrates that quasi-marketisation has resulted in the privatisatised responsibilities for both doctors and patients in healthcare assessment, care coordination, and financial management. The fourth section presents and analyses these findings, while the final section discusses their broader implications and offers policy insights for improving doctor–patient responsibility frameworks across diverse social and cultural contexts.

### Healthcare reforms in China and the changed doctor-patient responsibilities

Research on doctor–patient relationships and responsibilities has a lengthy history, researchers traced the study of doctor–patient relationships back to the medieval period [1]. The model proposed by Szasz and Hollender, comprising activity–passivity, guidance–cooperation, and mutual participation, laid the foundation for subsequent research on doctor–patient interactions [13]. Later studies suggested that consumerism and the rise of intelligent technologies have led to a redistribution of doctor–patient responsibilities, featured by more patient autonomy [3]. Collectively, these studies demonstrate that doctor–patient responsibilities are profoundly shaped by broader social context. Contrary to the belief that contemporary health services are marked by small scale, continuous change [14], China’s healthcare system has undergone three major reforms, each of which has brought corresponding changes to doctor-patient relationships and the distribution of responsibilities [15, 16].

After the 1950s socialist revolution, China established a unique intimate relationship between its people and the state through the direct allocation of resources. Most healthcare institutions in China were publicly owned and operated under district, municipal, and regional governments. The collective *danwei* (work-unit) system formed the foundation of primary care, providing free medical services and enabling bidirectional referrals across a three-tier structure of city hospitals, district or county hospitals, and work-unit clinics [17]. Doctors were salaried public servants without direct financial ties to patients, although undeclared payments by patients were said to be common, their primary duty was patient care [8, 18]. Thus, the government-dominated healthcare system, coupled with the ideology of collectivism, fostered a sense of organizational solidarity that far surpassed any divisions [7]. The doctor-patient relationship resembled paternalism as described in Western research [1, 13], and suggested that the principle of beneficence should take precedence over the principle of patient autonomy [3].

However, the state-funded healthcare system placed significant financial strain on the national budget [19, 20]. Beginning in the late 1970s and accelerating through the 1980s and 1990s, China implemented a series of economic reforms that introduced market mechanisms into public services, including healthcare [21]. By the turn of the century, the welfare-based system had been largely abandoned [21]. Starting in 1980 the Chinese government deliberately decreased its contribution to healthcare spending, which declined from 36.2% to 15.21% in 2002 [22]. Since the state provided only 6–8% of financial support for the budget deficits of hospitals, with the remaining approximately 90% of the shortfall being made up from revenues from medical services, and the sales of pharmaceuticals and medical devices [23]. Public hospitals were encouraged to generate their own income through service fees, drug sales, and operate with financial self-sufficiency and compete for patients and revenue. Doctors have shifted from being ‘national public service providers’ to practitioners in a healthcare market [7, 16].

At this stage, doctor-patient responsibilities in China have been transformed. As hospital revenues became directly tied to physicians’ income, doctors were compelled to align their practices with hospitals’ profit-oriented objectives and to derive part of their earnings from patient payments. Financial pressure transferred to patients. Nationwide, the annual per-capita cost of outpatient and inpatient care rose sharply by around 14% per year between 1993 and 2002, average outpatient costs increased from CN¥21.5 (UK£2.39) to CN¥99.6 (UK£11.07), while inpatient costs rose from CN¥933.4 (UK£103.71) to CN¥3,597.7 (UK£399.74). The phrase ‘hard to see the doctor, expensive to see the doctor’ became a common public complaint [24]. Marketisation, rather than improving healthcare services and communication, intensified distrust: patients increasingly questioned whether physicians’ decisions were motivated by care or by profit, leading to widespread scepticism about the ethical standards of medical professionals [25, 26].

The deterioration of doctor-patient trust and increasingly fierce conflicts have triggered the latest round of healthcare reforms, which are characterized by ‘quasi-marketisation’, combines state regulation with market incentives, maintaining government oversight while introducing competitive mechanisms to enhance efficiency [27, 28]. During this time, the three-tier healthcare system has been established, comprising primary healthcare facilities, secondary hospitals, and tertiary hospitals, purpose to manage institutions hierarchically according to their functions, service capacities, and technical levels, thereby achieving tiered diagnosis and treatment. Under this model, patients are expected to seek initial care at primary-level facilities and be referred upward as needed, improving the efficiency of medical resource utilisation and alleviating the problems of “difficult and expensive access to healthcare.” However, this system is not mandatory, and patients remain free to choose their preferred medical institutions [7, 21].

Hospitals increasingly rely on revenue generation for their existence, which concentrates health resources in economically developed regions and larger institutions [29, 30]. In 2023, the number of medical and health technicians per 1,000 residents reached 10.20 in urban areas and 6.55 in rural areas. The majority of healthcare resources are located in the eastern, more developed provinces [9]. The referral system remains weak: data from the Sixth Health Service Survey show that over half of two-way referrals fail to occur [31]. Tertiary hospitals, with superior staff and equipment, dominate the healthcare market and handle over 50% of all medical services [32, 33]. In addition, medical insurance reimbursement remains incomplete: patients are reimbursed only for part of their costs and continue to face rising out-of-pocket expenses. In recent years, annual medical costs have increased by around 8%. Between 2011 and 2021, the national average hospitalisation cost per visit rose from 6,632 yuan (£736.89) to 11,003 yuan (£1,222.50). The average outpatient cost grew from 180 yuan (£20.00) to 329 yuan (£36.56), marking an increase of about 83% [9].

Against this backdrop, doctor–patient responsibilities in China have undergone further transformation. Studies [7, 8, 27] suggest that doctor–patient relationships in contemporary China have increasingly taken on consumerist characteristics, although some research argues that elements of the traditional paternalistic model persist. This study contends that this quasi-marketised context provides a valuable case for examining how institutional norms shape the distribution of doctor–patient responsibilities.

### Institutional norms in healthcare

Healthcare systems function as a central institution in society, structured to maintain public health, support economic stability, and uphold social order [15]. Hayek et al., [34] considered institutions as shared societal expectations rather than explicit regulations. North described institutions as the frameworks governing social interactions, essentially the “rules of the game” in society [35]. These frameworks, which include both formal and informal societal norms and conventions, serve to structure everyday life and reduce uncertainty by establishing consistent patterns of behaviour.

Further developed in relation to healthcare, institutions are believed to have a high degree of resilience, and are legitimized by three major types of social forces, as Scott described as pillars: regulative, which involves laws, policies and contracts that dictate required actions; normative, which involves societal assumptions and expectations regarding appropriate actions; cultural-cognitive, which involves the implicit beliefs and mental frameworks that shape typical behaviours [15]. Although these aspects are always distinguished analytically, they often overlap in practice.

China’s healthcare system can be understood through the three institutional pillars. The regulative pillar is primarily shaped by policy directives from the Central Committee of the Communist Party and the State Council but has shifted from direct control to indirect governance. Hospitals are required to operate under self-financing principles and performance-based evaluation, while policy instruments such as insurance reimbursement, service pricing, and regional referral rules create an institutional environment that combines bureaucratic oversight with market competition, therefore confirming its quasi-market character [6]. Current research suggests that the existing healthcare system prioritises fairness, efficiency, and the protection of citizens’ health rights through a hybrid model that combines state leadership with market incentives [36]. It further argues that the normalisation of market logic has redefined health expenditure as a private investment rather than a collective responsibility [7, 33]. However, few studies have examined how these transformations, at the institutional level, shape the evolving distribution of doctor–patient responsibilities, leaving the gaps to explore how healthcare reforms restructure the responsibilities of doctors and patients.

In doing this, the paper addresses two main research questions:

1. How has China’s healthcare system reshaped the distribution of responsibilities between doctors and patients, and what implications has this shift had for their experiences?
2. What broader insights does the Chinese case provide for understanding how institutional norms shape the doctor–patient responsibilities?

## Methods

The study followed an Interpretive Description that aimed to explore the meanings and practices of doctor–patient responsibility within the broader institutional context of quasi-marketised healthcare. It adopted a flexible interpretive orientation that focuses on understanding the interplay between subjective meanings and institutional structures [37]. Thematic analysis was employed to identify recurrent patterns and interpretive themes across interviews with doctors and patients while remaining sensitive to the broader policy and social context of healthcare reform. This suited this study because it was aiming to richly describe an emerging phenomenon [38].

As a qualitative study, aimed at gathering views and experiences from doctors and patients on their responsibilities and experiences in the current healthcare system. Participants were purposively selected from 13 hospitals across five provinces to capture institutional and regional diversity. The sample included 10 public hospitals and three private hospitals, reflecting China’s healthcare resource distribution, where tertiary public hospitals dominate service provision [6]. Provinces were chosen to represent both economically developed and less-developed regions, encompassing eastern, central, and northern China. All participating doctors held valid Chinese practising certificates and had between two and twenty years of experience; most had over five years and direct exposure to institutional or managerial reforms. Patients were included if they had multiple healthcare encounters or long-term chronic disease management experience, enabling reflection on the perceived responsibilities of doctors and patients in the reformed healthcare environment. Particular attention was given to patients undergoing long-term treatment, as they were most directly affected by healthcare reforms.

Sampling continued until data saturation was reached, when no new conceptual insights or themes emerged [39]. Thematic saturation occurred after interviews with the 11 doctor and the 13 patient, with two additional interviews conducted to confirm stability of the patterns. A total of 28 semi-structured interviews were conducted with 13 doctors and 15 patients, as detailed in Table 1 Study Participants:

**Table 1:** Study participants

Group	*n*	Professional level / condition type	Yeas of experience / illness duration	Region and hospital type
Doctors	13	• Junior (4) • Middle (5) • Senior (4)	• 2–5 years (5) • More than 5 years (8)	• 10 public hospitals (6 tertiary, 2 secondary, 2 primary) • 3 private hospitals across five provinces
Patients	15	• Multiple healthcare experience (more than five visits) (8) • Long-term management of chronic conditions (7)	• Short-term visit (8) • 3–5 years (2) • More than 10 years (5)	• From five provinces representing both economically developed and less-developed regions

Before each interview, participants received an information sheet outlining the study’s aims and procedures, along with a consent form. Interviews began only after written consent was obtained. This step is crucial for maintaining transparency and ensuring that participants are fully aware of the nature of the research and their role in it. Semi-structured interviews were conducted in Mandarin using a hybrid format of online (through Tencent Meeting, a widely used platform in Mainland China) and face-to-face sessions. The interviews followed an open-ended structure guided by a set of core questions exploring professional background, perceptions of responsibility, and experiences within the healthcare system. The duration of interviews ranged from 40 min to two hours. All interviews were audio-recorded, with supplementary notes taken as necessary.

All interviews were conducted and transcribed in Mandarin by the primary researcher. Key excerpts and coded segments relevant to theme development were translated into English by the same researcher, who is fluent in both Mandarin and English. To ensure translation accuracy and conceptual equivalence, one full transcript was independently reviewed by co-researcher. Minor inconsistencies were discussed and resolved through consensus. Selected translated excerpts were further checked against the original Mandarin text during analysis to preserve participants’ intended meanings and enhance the validity of cross-language interpretation.

Thematic analysis followed structured and iterative steps. The primary researcher conducted initial coding in Chinese to ensure linguistic accuracy and minimise translation bias [40]. A codebook was developed inductively through repeated readings and refined in consultation with two supervisory experts. Key coded segments and representative extracts were translated into English and regularly discussed with the supervisory team to maintain interpretive consistency. All discrepancies were resolved through discussion, and consensus was reached on the final themes, enhancing the credibility and transparency of the analytic process. Finally, the three themes: privatised responsibility in accessing care, care coordination, and medical financing, were established to the main findings.

The primary researcher, a native Mandarin speaker with professional training in sociology and prior experience in China’s healthcare sector, conducted all interviews and data analysis. This linguistic and cultural familiarity facilitated rapport with participants and accurate interpretation of meanings. The second researcher, an English-speaking professor with expertise in qualitative methodology, provided external oversight and reviewed translated extracts to ensure interpretive consistency and minimise potential bias. Reflexive notes and regular supervisory discussions further enhanced the transparency and balance of the analytic process.

To protect the privacy and confidentiality of the participants, each doctor and patient was assigned a code: Dn (*n* = 1 to 13) for doctors and Pn (*n* = 1 to 15) for patients. For sections that could potentially identify participants, identifiable information has been marked with (*) and supplemented with descriptive content in brackets to preserve the sentence’s full context and meaning. The study was approved by the University of Sheffield Research Ethics and Integrity (Version 8.3 – October 2024). All participants provided informed consent. The study recognises its limitation in representativeness, as the views expressed may not fully capture the perspectives of all healthcare staff or patients.

## Findings: privatised responsibilities of doctors and patients

### Privatised responsibility in accessing healthcare

The patients interviewed reported freedom to access any healthcare institution and being entirely responsible for their choice. One patient shared her experience of choosing a hospital.

Yes, it’s about making my own choice, because I can go anywhere. If it’s just a cold or fever, I might go to a small hospital or even a pharmacy to get some medicine. If it feels more serious, or I’m not sure, or if I’m not confident handling it myself, I go to a big hospital for peace of mind. (P1)

As patients could choose any healthcare institution directly without initial registration, they were free to prioritize top-tier hospitals. As one said:

I would definitely choose a large hospital first; small clinics are unreliable. (P15)

The influx of patients has greatly increased the workload of doctors in tertiary hospitals. Many interviewees described feeling overwhelmed by the sheer number of patients they must see each day, which limited their ability to meet patients’ expectations. One doctor noted that the heavy workload in his clinic severely curtailed the time available for each consultation, thereby compromising the quality of care.

I must see 30 to 40 patients in a half day clinic, and the consultation time for each patient may be five to ten minutes… there will be a lot of surgeries, and I will be very busy, I do not have much time to think of the patients’ feeling or comfort them when they feel sad. (D5)

In smaller cities and hospitals, the scarcity of medical resources means that some conditions cannot be effectively treated locally, compelling patients to seek medical care in larger cities with more advanced healthcare facilities. This phenomenon is described in China with a specific term, ‘yi di jiu yi,’ which refers to medical treatment and drug purchase activities at medical institutions outside the insured area [41]. Interviews were conducted with patients who have experienced seeking medical care in different locations. One such patient described her experiences with seeking medical care in another city, noting that it presented significant challenges because she needed to consider multiple factors, including medical information, hospital data, insurance, and living arrangements, thereby revealing a more comprehensive scope of patient responsibilities.

My hometown is in a small city, few hospitals know about my issues, so I went to four or five hospitals across different cities… At first the hospital in my hometown didn’t give one, said it wasn’t very clear. I kept asking around, searching information myself, different hospitals have different requirement, I checked my insurance, and where to eat food, is basic, but necessary. (P3)

After the patients decide which hospital and department they need to visit, they must select a specific doctor (if applicable), consultation time, and sometimes the level of expertise (this step in Chinese is called: gua hao). Based on patient descriptions, the responsibility for choosing a doctor during the registration process also falls on the patients. One shared his strategies for selecting a doctor during the registration process, which included his consideration on assessing whether the health condition matches the scope of the consultation with the registered doctor, the doctor’s specialization and experience, and availability.

The process is pretty similar in most hospitals, in that you have to register as the first step. But how to choose an appropriate doctor, right? It’s very tricky… you can’t just register with a specialist, because they may be busy, many appointments, you have to wait for a long time, and you may recover by yourself when you get an appointment. But you also can’t just say, register with a random one. I would check online. (P14)

To avoid long waiting times and gain faster access to healthcare services, a practice of ‘relationship-based care’ has emerged. This approach involves patients using their personal connections and acquaintances to obtain a more favourable position in accessing medical treatment and establishing doctor-patient relationships. A patient recounted his experience of getting in touch with a doctor as,

I reached a famous doctor through (a personal relationship*), otherwise I couldn’t get that appointment…It is very common to use personal relationships to find a doctor. (P15)

Another patient also mentioned relationship-based care. Her account further revealed the purposes behind this behaviour: on one hand, to gain quicker access to medical resources, and on the other, to ensure a better healthcare experience.

I will contact my doctor friends, I often use acquaintances as a chain of medical experience, will be able to see the doctor more quickly, in addition, part of it, the doctor will be because I was referred by an acquaintance, the whole experience will be a little better. (P14)

Thus, the privatisation of responsibilities in accessing healthcare appears to have transformed the way patients access healthcare.

### Privatised responsibility in care coordination

The research revealed that both doctors and patients currently bear responsibilities in care coordination. This typically involves patient navigation within hospitals, assessing and updating care plans, managing referrals to appropriate hospitals, and coordinating post-treatment.

One doctor participant reflected that his current tasks include initial assessment of a patient’s condition and was responsible for making subsequent treatment decisions tailored to the patient’s actual situation.

The patients come here on their own registration, so as a doctor, the first thing I have to do is to judge, whether he should have registered with me or not. I need a preliminary judgement. Then sometimes when I listen to his description and think, for example, he has (a symptom*), then I have to make a further judgement and may have to send him for some tests. (D11)

This doctor then explained the tasks he undertook during the navigate process, which include administrative load and required him to spend more time coordinating care for patients.

Some patients just come to the wrong place, registered wrong, so what to do, they are already here, I cannot directly let people go, I will need to explain, you came to the wrong place, I’m not here to see this disease, you go to another building, go to which department, you go to register that. (D11)

The interviews revealed that patients increasingly assume decision-making responsibilities and are required to evaluate the potential consequences of their own care plans.

The doctor gave me two choices, it’s okay to treat it with him, but his treatment plan is to apply medication and treat it more conservatively. However, he also told me that I can go to (a hospital name*), where they can directly operate, and it will be faster. It’s my choice. I’m thinking about the hassle of referrals and the fact that I have to go through all these processes all over again, so I’ve been evaluating the pros and cons. (P14)

Patients reported being responsible for tracking their own medical records, referrals, and related documents to ensure continuity of care. In a healthcare system with limited collaboration and information sharing, they often had to maintain records themselves. Several patients also noted that transferring between institutions frequently required repeat tests due to poor data integration.

I kept the receipts from each inspection. Records, diagnoses, all that. Because I might need them next time. If I get referred to another hospital, I may need another checkup, too, generally speaking, I’ll need to. Because they don’t share with each other. (P14)

For patients, this situation also signified that they have taken on increased responsibilities in managing navigation and referral, needing to advocate more actively for themselves and seek information about the referral pathway.

I must register and get into this hospital first and then figure out what to do. So, it might have been that the department that I got registered in wasn’t the right one to begin with, so I went through a series of referrals, these later on. The process is, you keep going around inside the hospital, just listening to the doctors, or just asking around on your own. (P13)

The privatisation of assessment and care planning responsibilities leaves both doctors and patients feeling vulnerable, wishing to reduce the responsibilities they bear in doctor-patient interactions and hoping that the other party takes on more responsibilities. As one doctor summarised,

I will go ahead and follow the standard, the standard process of this treatment. But give advice to the patient, like, saying, where do you should go next. This is not good for me. You do not know what people will think. And there’s a lot of things that need to be explained, and sometimes it’s hard to explain, so, I’ll just, make and get the standard treatment good. (D9)

Interviews with patients indicated that some doctors sought to reduce their decision-making responsibilities. One patient reported that during the development of her care plan, doctors repeatedly shifted decision-making to her, placing the burden of choice on the patient.

I did not know how long I should stay in the hospital, which made me very anxious. I went to ask the doctor, and he said, ‘depend on you’. Then I asked if I should do another CT scan to check the brain and he responded, ‘you can have it whenever you want’. I was really confused. I’m not a doctor; I don’t have medical knowledge. How can I be expected to make all these decisions? (P3)

These extracts reveal the range of responsibilities that doctors and patients assume in care coordination, including care planning, assessments, referrals, and follow-ups. The analysis further shows that both parties feel vulnerable in taking on these responsibilities and often seek to avoid or shift them, reflecting a sense of uncertainty and fragility in the current allocation of care duties.

### Privatised financing of medical cost

The current healthcare system transferred the responsibility for medical costs to individual patients. Currently, although the release of Opinions on Deepening the Reform of the Medical Security System by the State Council in 2020 aimed to enhance people’s welfare [28], a significant portion of medical expenses still falls directly onto patients [42], creating for some a financial burden. This extract from one patient interview illustrates the personal cost associated with treatment.

Health insurance didn’t cover much. Well, the first treatment cost was reimbursed, but I required several visits, the first one was fully covered, but after that, they only reimbursed about 100 yuan, in the following visits, the amount they covered became less and less. (P15)

This patient indicated that complex reimbursement requirements are a major barrier to patients claiming their expenses. Partial reimbursements often failed due to complicated procedures, resulting in patients bearing the costs themselves. This patient added,

These clauses are complex, sometimes I’m not sure if I can get reimbursement, maybe I’m lazy or busy, I’ll miss the time to get the money back. (P15)

In some cases, patients must bear the full cost of medications and treatments which are not covered by health insurance, placing the financial burden directly on individuals. This situation poses significant barriers for those lacking financial resources, as they must find ways to fund their healthcare needs independently. A patient described his experience of paying out of pocket for an injection, showing the strain that medical costs put on him.

Each dose cost about 400 or maybe 500 yuan. I needed 6 doses every day, and it would be over 3,000 yuan. But I was running out of money. Sometimes, I could only get injection if I managed to borrow money in the morning. If I had money that day, I would come to the hospital to pay and then I could get the injection. Undergoing (a disease*) is really tough. You see in TV shows, someone said, ‘doctor, no matter how much it cost, I don’t care, I just want the patient to live.’ That’s not true, reality is cruel. Many fellow patients genuinely cannot afford treatment and end up giving up. I have seen this very clearly. (P6)

The privatisation of medical costs has undoubtedly placed financial pressure on patients. One patient who was hospitalized, expressed dissatisfaction with the medical expenses,

I was really anxious, as I lived in hospital for 7 days, money went by so fast for around two to three thousand RMB every day. (P3)

A doctor indicated that the privatisation of financial responsibilities was one of the main factors transforming the current doctor-patient relationship into a consumer-purchaser relationship. This shift turns the doctor-patient encounter into a transactional relationship, leading patients to view treatment outcomes as purchasable goods, without considering the many uncontrollable factors that can affect these outcomes.

If the patient thinks, I’m the one who paid a lot of money to come to the doctor, I’m the one who came to buy your services, I spent money, I spent so much money, I should get better. (D2)

Privatised responsibility also puts doctors in a difficult position, caught between patients’ financial constraints and the hospital’s fiscal pressures. Interviews with doctors revealed their awareness of the issue as a societal problem, which is challenging to address at the interpersonal level. Confronted with their patients’ financial hardships, this doctor felt ‘helpless’ because, he was unable to make decision for the patients, nor could he compensate for the deficiencies in the healthcare system.

This is a deeper social problem, I know some patients fall into financial hardship, they even sell all of their properties, even if the disease is cured, how can they live then if they have nothing. But for me, a doctor, shall I ask them not to treat, or I should say nothing but respect their choice, I do not know which one is good, I’m just doing what a doctor could do. (D6)

The dilemma doctors now face is entirely the construction of an ideologically driven policy. The explicit aim of the state’s retreat from public hospitals was to enhance service efficiency, enabling hospitals to be self-sustaining and financial independent from state funding. In line with this objective, the burden of generating additional income for hospitals has shifted to doctors. The extract below emphasizes that medical marketisation has not disappeared but has been further consolidated and, indeed, has gradually intensified the competitive environment, placing additional pressure on doctors.

The problem with hospitals is that they still aim to be profitable, as there’s no motivation without profit. In recent years, X hospital has suffered significant losses, unable to pay salaries to its staff. Therefore, it (the hospital*) requires us (the doctors*) to find ways to generate revenue and increase profits. They set performance indicators, and, if doctors don’t meet the standard, it’s up to hospital to figure out a solution. (D2)

Therefore, the quasi-marketised healthcare system has transferred part of the financial burden to individuals, making patients’ ability to pay a decisive factor in accessing essential medications and equitable medical care.

## Discussion

This study set out to address two questions, first, to identify has China’s quasi-marketised healthcare system redefined the distribution of responsibilities between doctors and patients, and what implications has this shift had for their experiences and interactions; and second, to understand how institutional norms shape doctor–patient responsibilities.

Our data suggests that China’s healthcare system has redefined responsibility through three forms of privatisation: accessing healthcare, care coordination, and healthcare financing, which have transferred systemic and administrative duties to individuals. These changes have increased the pressures placed on both doctors and patients, generating new forms of vulnerability, mutual mistrust, and dissatisfaction within clinical encounters. Using Scott’s institutional framework, the study explored the way that privatised responsibility is normalised and legitimised. The regulative pillar decentralises responsibility through market-oriented reforms; the normative pillar redefines professional and moral expectations; and the cultural–cognitive pillar embeds market rationality as common sense. These influences are not independent but interact with and reinforce one another.

Under the regulative pillar, state control establishes the bureaucratic structure of the healthcare system; however, market incentives within it enable medical institutions to operate with a considerable degree of autonomy, ^7^ and increasingly required patients to take personal responsibility for accessing healthcare services. This arrangement contrasts with many Western healthcare systems (e.g. in the UK), where gatekeeping or primary care system structure patient flow. The lack of an organised referral system in China means that some patients travel across regions to obtain care, reflecting the privatisation of access responsibilities. Moreover, patients must also select their doctors, yet popular and well-regarded physicians are often overburdened with demand. Privatised responsibility in accessing healthcare drives the phenomenon of ‘relation-based care’. To reduce waiting times or secure perceived higher-quality treatment, patients commonly rely on personal or family connections to facilitate appointments.

This phenomenon brings the change of cultural–cognitive pillar which involves the taken-for-granted beliefs and interpretive frameworks. Market logic has become internalised as common sense: firstly, market incentives transform doctors into “commodities” within the healthcare market. From a patient’s perspective, a doctor’s skills are seen as valuable resources. Experienced doctors, therefore, become scarce commodities over which patients compete [19, 21]. Secondly, patients’ attempt to build personal relationships with doctors through financial incentives, hoping to secure superior care. This approach seeks to convert monetary gifts into preferential treatment, blending economic transactions with doctor-patient relationships and challenging the fairness of the healthcare system [43].

The shift to quasi-marketisation produce the change in the normative pillar. As the current healthcare system has transferred many administrative duties and navigational duties, previously managed through institutional referral systems, onto individuals, professional norms were redefined to align with performance-based incentives and institutional efficiency. This has profound implications for both doctors and patients.

For doctors, the privatisation of coordination, navigation and referral responsibilities requires additional time for communication, providing emotional support to patients, documentation, and follow-ups. These additional duties often compete with doctors’ clinical responsibilities and increase their workloads. For patients, privatised responsibilities in coordination and decision-making for follow-up healthcare management are limited by their health literacy [44]. Patients with limited understanding of medical terms and healthcare procedures are disadvantaged as they may receive suboptimal care or fail to complete treatment plans.

In this system of privatised responsibility, both doctors and patients experience vulnerability and seek to shift decision-making to the other party. Doctors may deflect responsibilities to avoid the risks of medical complaints arising from miscommunication or unmet expectations, while patients, lacking adequate knowledge and institutional support, struggle to navigate complex care pathways. This reciprocal uncertainty weakens mutual trust and contributes to dissatisfaction within the doctor–patient relationship [45]. Consequently, both doctors and patients often feel unsupported by the healthcare system, which has fragmented accountability and intensified strain on both sides.

The current payment system has also shifted healthcare financing responsibilities towards privatisation. Despite reports that 95% of the population was covered by National Medical Insurance by 2020 [46], substantial challenges persist due to limited reimbursement rates and the narrow scope of the services covered. Personal and family income largely determine access to care: higher-income groups can afford better and timelier treatment, while the lower-income ones face restricted access and heavier financial burdens.

Market logic has become normalised as a taken-for-granted understanding of healthcare, compelling hospitals to pursue financial sustainability through profit generation. The ability to pay, choose, and manage one’s care is increasingly seen as a marker of personal responsibility and moral worth. This change transforms doctor–patient interactions into transactional relationships and commodifies treatment outcomes. Patients, in turn, engage with healthcare services through consumerist expectations, seeking choice, immediacy, transparency, and measurable results which often clashing with the traditional ethos of trust and professional authority [7]. Moreover, this privatisation of responsibilities also requires doctors within the current healthcare system to balance hospital profitability with patient financial burdens, which is likely to affect decision making and strain doctor-patient relationships: while patients naturally seek to minimize their healthcare expenditures, doctors are compelled to focus on profitability to sustain their practices and support their hospitals. As previous research has shown, economic pressures and moral dilemmas contribute to ethical burden on doctors [4, 7].

This study further argues that institutional norms are continually reinterpreted through the daily practices of doctors and patients. In navigating care pathways, negotiating treatment decisions, and managing financial pressures, the institutional logics have been redefined. In this sense, the institutional pillars do not simply determine responsibility; they are themselves sustained and reshaped through the ongoing interactions and negotiations between doctors and patients.

## Conclusion

The findings reveal that the doctors-patient responsibility has be privatised in China. The findings call for renewed attention to how institutional arrangements shape and redistribute doctor–patient responsibilities, reflecting both the complexity of systemic transformation and the inequalities it may reinforce. The empirically grounded use of institutional theory proposed here holds potential for wider application in analysing transitions in health service governance and further study of healthcare transformation.

## Limitation

Although our research examines the quasi-marketisation in healthcare reshapes doctor-patient responsibilities and highlights its potential negative impacts, offering a comprehensive alternative prescription lies beyond its scope. Nonetheless, the pilot nature of the research and findings is readily acknowledged. Future research should engage with larger more representative populations and consider focusing on the responsibilities and challenges faced by patients with specific diseases within the healthcare system.

## Data Availability

The datasets generated and/or analysed during the current study are not publicly available due to the potential risk of identifying participants from interview content, but are available from the corresponding author on reasonable request.
